# Identification and validation of the clinical roles of the VHL-related LncRNAs in clear cell renal cell carcinoma

**DOI:** 10.7150/jca.55113

**Published:** 2021-03-05

**Authors:** Wuping Yang, Jingcheng Zhou, Kenan Zhang, Lei Li, Yawei Xu, Kaifang Ma, Haibiao Xie, Lin Cai, Yanqing Gong, Kan Gong

**Affiliations:** 1 Department of Urology, Peking University First Hospital, Beijing 100034, P.R. China.; 2 Hereditary Kidney Cancer Research Center, Peking University First Hospital, Beijing 100034, P.R. China.; 3 Institute of Urology, Peking University, Beijing 100034, P.R. China.; 4 National Urological Cancer Center, Beijing 100034, P.R. China.

**Keywords:** VHL, lncRNAs, ccRCC, FGD5-AS1, survival

## Abstract

Accumulating evidence suggests that lncRNAs (long non-coding RNAs) function as oncogenes or tumor suppressor genes in ccRCC (clear cell renal cell carcinoma). Although VHL (Von Hippel-Lindau) gene inactivation is by far the most common carcinogenic driving event in ccRCC, the roles of VHL-related lncRNAs in ccRCC remain unknown. In this study, using RNA-seq and clinical data in TCGA-KIRC (the Cancer Genome Atlas-Kidney Renal Clear Cell Carcinoma), we identified VHL-related lncRNAs through WGCNA (Weighted Gene Co-expression Network Analysis), correlation analysis and catRAPID algorithm, and explored their clinical characteristics in ccRCC. Results showed that 10 lncRNAs (AC112220.2, AL391121.1, USP46-AS1, AL450326.1, MID1IP1-AS1, SUCLG2-AS1, RAP2C-AS1, FGD5-AS1, AC018647.2 and AC015922.2) were identified as VHL-related lncRNAs, and they were down-regulated in ccRCC tissues. Survival analysis results indicated that high expression groups of AC112220.2, AL391121.1, USP46-AS1, AL450326.1, SUCLG2-AS1, RAP2C-AS1, FGD5-AS1, AC018647.2 and AC015922.2 had significantly longer OS (Overall Survival) than their respective low expression groups. Meanwhile high AC112220.2, USP46-AS1, AL450326.1, SUCLG2-AS1, FGD5-AS1, AC018647.2 and AC015922.2 expression groups had remarkably longer DFS (Disease Free Survival) than their respective low expression groups. Besides, FGD5-AS1 and AL391121.1 expression were decreased in VHL mutant tissues compared with VHL non-mutant tissues. Moreover, high expression group of FGD5-AS1 had significantly longer OS and DFS than their respective low expression groups in VHL mutant ccRCC. In addition, we found that DNA hypermethylation may also play an important role in decreased FGD5-AS1 expression. Furthermore, we validated the expression of FGD5-AS1 in VHL mutant and non-mutant ccRCC tissues and cell lines. In conclusion, our results demonstrated that lncRNA FGD5-AS1 was significantly associated with VHL and can serve as a novel biomarker of ccRCC.

## Introduction

RCC (Renal cell carcinoma) is the second leading cause of death in human urological malignancies, accounting for 2-3% of all cancer diagnoses and cancer deaths worldwide 1. The global burden of RCC is approximately 403,262 new cases and 175,098 deaths per year, with an annual rate of increase of approximately 0.7% 2. The most common histologic subtype ccRCC (clear cell RCC) represents about 75-80% of RCC, and up to 92% of these cancers have inactivated the VHL (Von Hippel-Lindau) gene 3. Partial or radical nephrectomy represents the clinical protocol for localized RCC, and the 5-year cancers specific survival rates for patients with localized RCC (pathological stage pT1-2) after nephrectomy ranges from 71% to 97% 4, 5. For patients with locally advanced tumors, 5-year cancer-specific survival rates after nephrectomy decrease to 20% to 53% 4. Despite nephrectomy with curative intent, about 30% of patients with ccRCC with localized disease eventually develop metastases, and the 5-year survival rate of metastatic ccRCC dramatically drops down to 10% 3, 6. Therefore, finding promising early detection markers and more effective therapeutic targets is imperative to improve the prognosis of ccRCC patients.

As a new class of ncRNAs (non-coding RNAs), lncRNAs (long ncRNAs) are characterized as non-coding transcripts greater than 200 base pairs in length transcribed by RNA Pol II from independent promoters 7. In many cases, lncRNAs have been proven to be the main regulator of gene expression and thus, they can play a key role in a variety of biological functions and disease processes including cancers 8. For example, lncRNA LBCS inhibits castration resistance of prostate cancer 9; DANCR promotes cell metastasis and proliferation in bladder cancer 10. Besides, recent accumulating evidence has indicated that lncRNAs, such as OTUD6B-AS1, URRCC, HOTAIRM1 and MRCCAT1, play important regulatory roles in diverse biological processes in ccRCC 11-14. As for the molecular characteristics of RCC, VHL gene inactivation is by far the most common carcinogenic driving event in ccRCC 15. However, there are currently few studies on the roles of VHL-related lncRNAs in ccRCC. Therefore, it is urgent to identify VHL-related lncRNAs and understand their roles in ccRCC.

In the present study, using RNA-seq and clinical data in TCGA-KIRC cohort, we identified VHL-related lncRNAs by WGCNA, correlation analysis and catRAPID algorithm, and explored their prognostic value and relationship with clinicopathological characteristics in ccRCC.

## Materials and Methods

### Ethics statement

This study was approved by the Biomedical Research Ethics Committee of Peking University First Hospital (Beijing, China, IRB00001052-18004). Written informed consents were also obtained from all patients.

### TCGA and GEO data

Level-3 RNA-sequencing data, the clinicopathological and survival data of patients with ccRCC were downloaded from TCGA (https://portal.gdc.cancer.gov/). Briefly, 539 ccRCC and 72 adjacent normal renal tissues were included in this study. Their clinical and survival data, including tumor stage, lymph node, metastasis, pathological stage, histologic grade, OS (Overall Survival) and DFS (Disease Free Survival), were downloaded. The DNA methylation data of 325 ccRCC and 160 adjacent normal renal tissues were also downloaded. In addition the GSE105260 dataset which contained the methylation data of 485512 CpG sites in 9 normal kidney, 9 primary ccRCC and 26 metastasis ccRCC tumors was obtained from GEO datasets (https://www.ncbi.nlm.nih.gov/geo/query/acc.cgi?acc=GSE105260).

### Weighted Gene Co-expression Network Analysis (WGCNA)

The R software package “WGCNA” was used for weighted gene co-expression network analysis. It is an algorithm for constructing co-expression networks, defined by the similarity of gene co-expression. Briefly, we first used paired Pearson correlations to evaluate the weighted co-expression relationships among the subjects in all data sets in the adjacency matrix. Then, a topological overlap matrix (TOM) similarity function was used to convert the matrix to a TOM. The resulting TOM was based on genetic similarity of biological significance and was used to measure the co-expression relationships between genes, and the defined weight threshold was 0.02.

### catRAPID

catRAPID (http://s.tartaglialab.com/page/catrapid_group) was an algorithm to estimate the binding propensity of protein-RNA pairs. By combining secondary structure, hydrogen bonding and van der Waals contributions, catRAPID predicts protein-RNA associations with great accuracy.

### Clinical samples and ccRCC cell lines

The paired tissues samples (adjacent normal renal tissue and ccRCC tissue) from 54 ccRCC patients including 12 ccRCC caused by VHL mutation were collected from Department of Urology, Peking University First Hospital for total RNA extraction, reverse transcription and q-RT-PCR analysis. The total RNA of HEK293, OSRC2, ACHN, A498, 769-P and 786-O was also extracted for reverse transcription and q-RT-PCR analysis. The detailed primer sequences was provided in Supplemental Table 1.

In addition, pLV-hef1a-mNeongreen-P2A-Puro-WPRE-CMV-VHL-3Xflag and its corresponding control plasmid vector were constructed by the SyngenTech Company (SyngenTech Co. Ltd., Beijing, China). Cells were transfected with the corresponding vector using Lipofectamine 3000 Transfection Reagent (Invitrogen, USA) according to the manufacturer's instructions. Lenti-virus was produced using three vectors system: transfer vector, viral packaging (psPAX2) and viral envelope (pMD2G) at 6:3:1 ratio transfected into 293T cells. VHL-overexpressed stable cell lines were selected with puromycin (5 μg/mL). The total protein and RNA of VHL-overexpressed 786-0 cells and the control 786-0 cells were extracted for western blot and q-RT-PCR analysis. For western blot assay, protein (30 μg per lane) was separated by SDS-PAGE and then were transblotted to PVDF membranes, and membranes were blocked in 5% nonfat milk powder and incubated overnight at 4 °C with anti-Flag (1:1000; CST, 14793S), anti-VHL (1:1000; CST, 68547S) and anti-GAPDH (1:10000, Proteintech, China). After incubated with horseradishperoxidase-conjugated goat anti-rabbit IgG, membranes were resolved by chemiluminescence.

### Statistical analysis

Non-parametric Mann-Whitney test was used to detect differences in continuous variables. The Pearson's correlation test was conducted to assess the correlations between lncRNAs and VHL. The prognostic roles of VHL co-expressed lncRNAs were examined with the Kaplan-Meier method, and the log-rank test was conducted to determine the significance of the difference between the survival curves. A P-value < 0.05 represented statistical significance. The statistical analyses were all carried out by R Studio and GraphPad Prism 7.00.

## Results

### Identification of VHL-related lncRNAs in ccRCC

**Figure 1:**
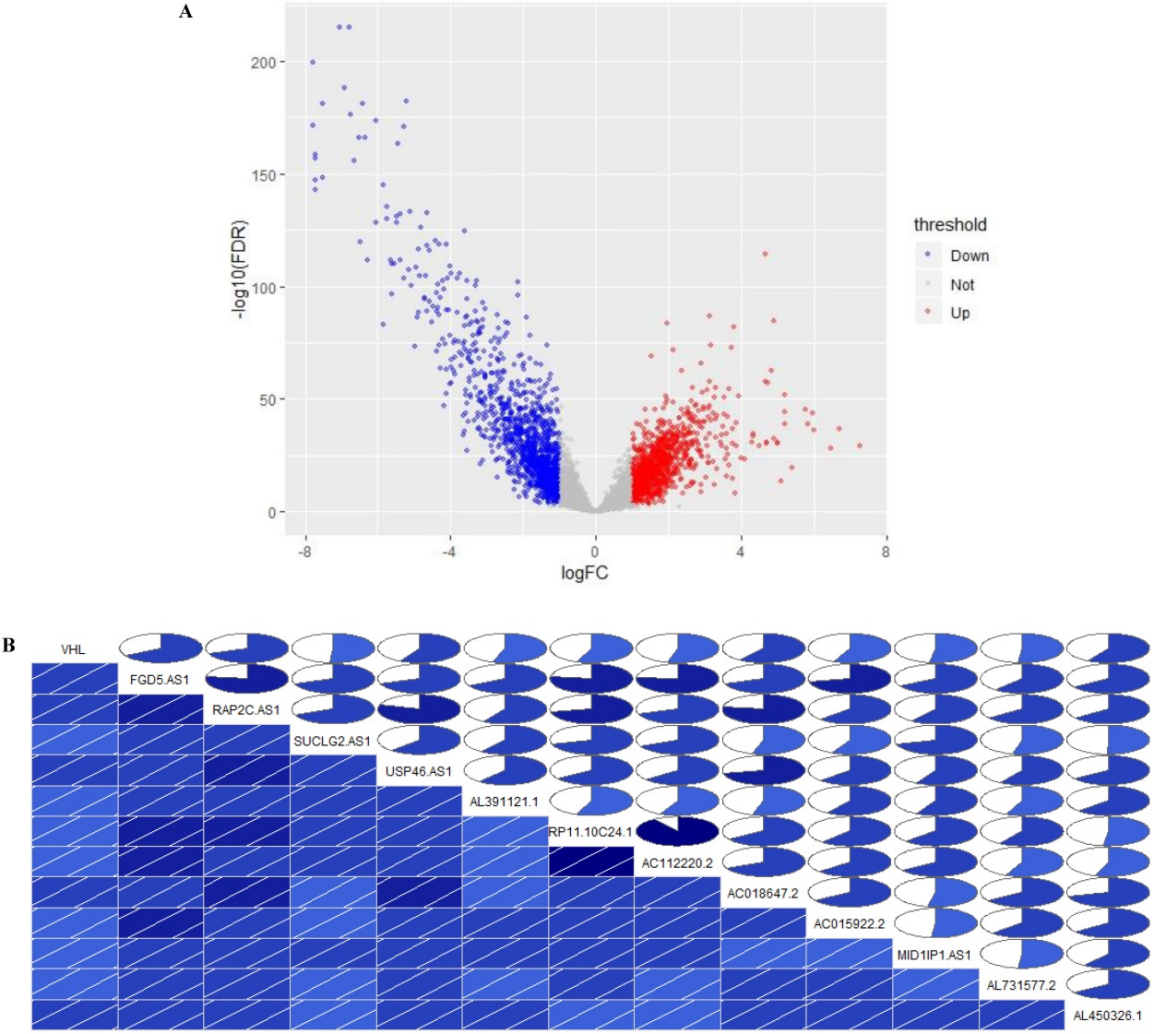
** Identification of VHL co-expressed lncRNAs.** (A) Volcano Plot, (B) The correlation between the expression of 12 lncRNAs and VHL. In the lower triangular cell, the blue color and the slash from lower left to upper right indicate that the two variables in the cell are positively correlated. The darker the color, the higher the saturation, and the greater the correlation between variables. Similarly, the upper triangle cell displays the same information in a pie chart. The color function is the same as above, and the degree of correlation is displayed by the size of the filled pie chart.

**Figure 2:**
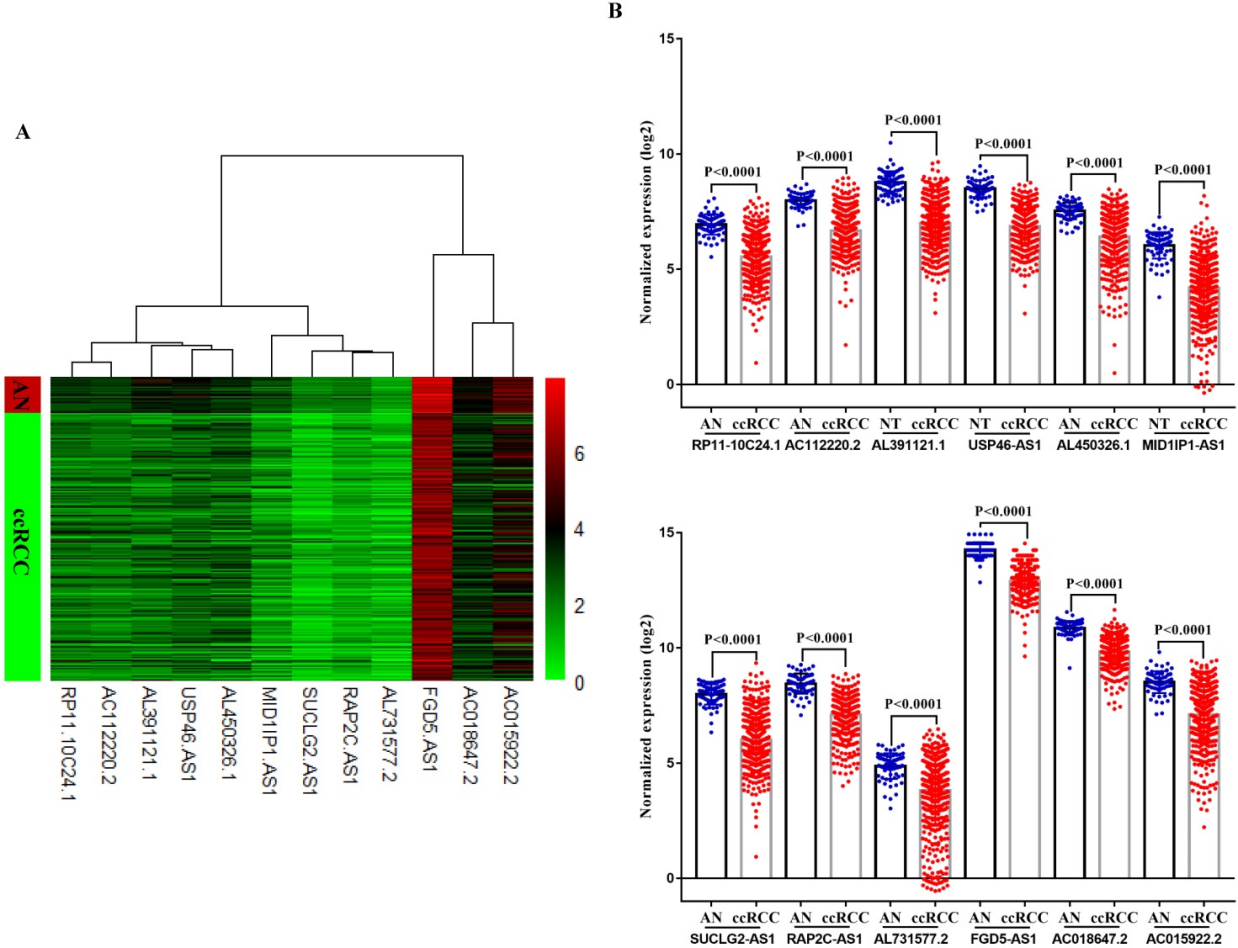
** Comparison of VHL co-expressed lncRNAs expression in ccRCC tissues and adjacent normal tissues.** (A) Heat map (B) Statistical comparison.

**Figure 3:**
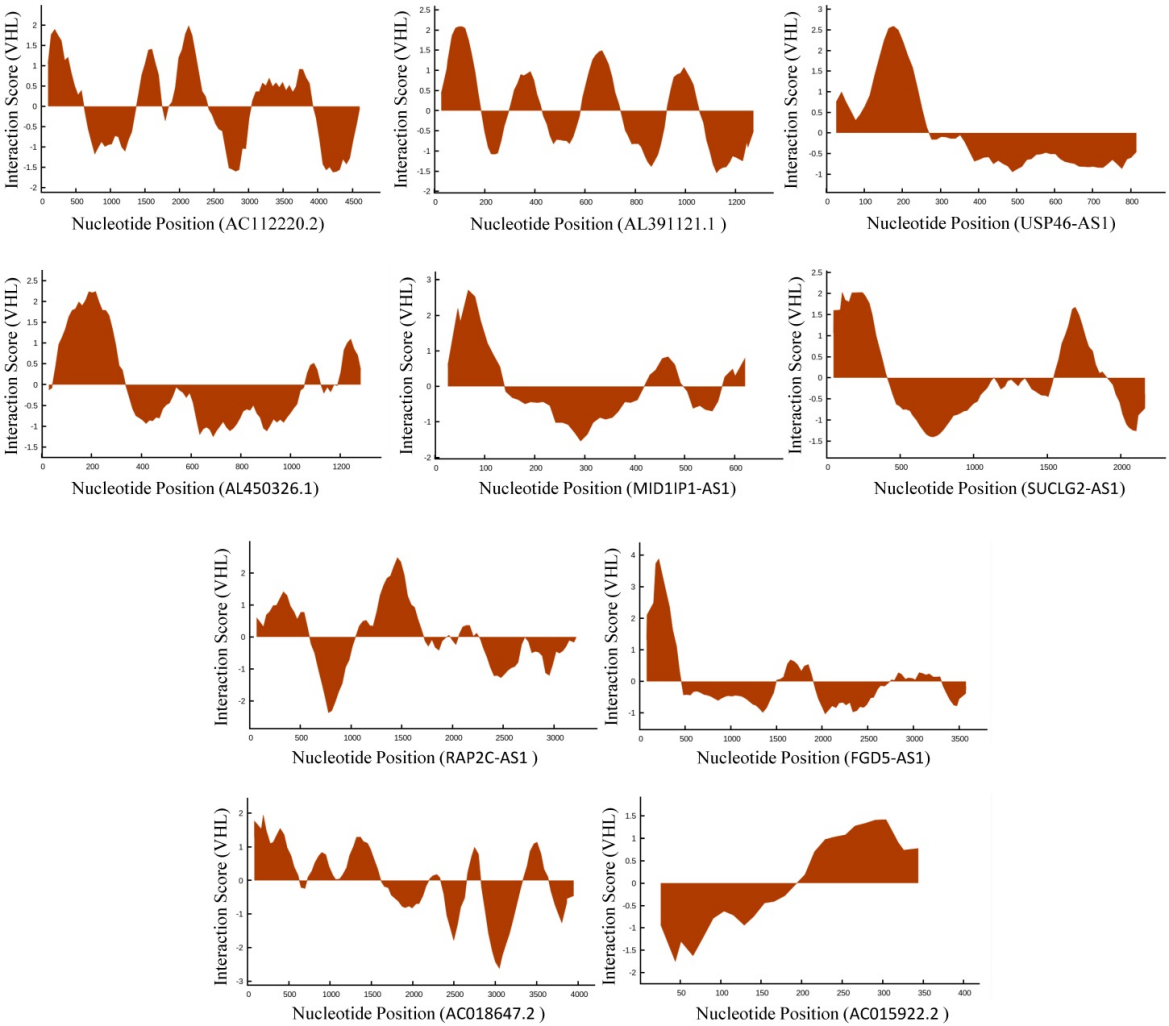
Estimation of the binding propensity of VHL protein and lncRNAs.

In TCGA-KIRC cohort, tumor tissues from 539 cases of ccRCC were subjected to RNA-seq study, among which 72 cases had matched adjacent normal tissues. The differential analysis was performed by “edgeR: a Bioconductor package for differential expression analysis of digital gene expression data” algorithm, results showed that 1333 lncRNAs were down-regulated and 1364 lncRNAs were up-regulated in ccRCC tissues compared to adjacent normal tissues (Figure 1A). Then the expression data of these 2697 lncRNAs and VHL was performed to WGCNA, results showed that 12 lncRNAs (FGD5-AS1, RAP2C-AS1, SUCLG2-AS1, USP46-AS1, AL391121.1, RP11-10C24.1, AC112220.2, AC018647.2, AC015922.2, MID1IP1-AS1, AL731577.2 and AL450326.1) were co-expressed with VHL (Table 1). Correlation analysis results showed that all of these 12 lncRNAs were highly positively correlated with VHL (Figure 1B). Moreover, all of these 12 lncRNAs were down-regulated in ccRCC tissues compared to adjacent normal tissues (Figure 2A and 2B). Furthermore, we used catRAPID to estimate the binding propensity of VHL protein with these 12 lncRNAs, and our results showed that AC112220.2, AL391121.1, USP46-AS1, AL450326.1, MID1IP1-AS1, SUCLG2-AS1, RAP2C-AS1, FGD5-AS1, AC018647.2 and AC015922.2 have a certain binding potential with VHL protein (Figure 3).

### Prognostic value of VHL-related lncRNAs in ccRCC patients

**Figure 4:**
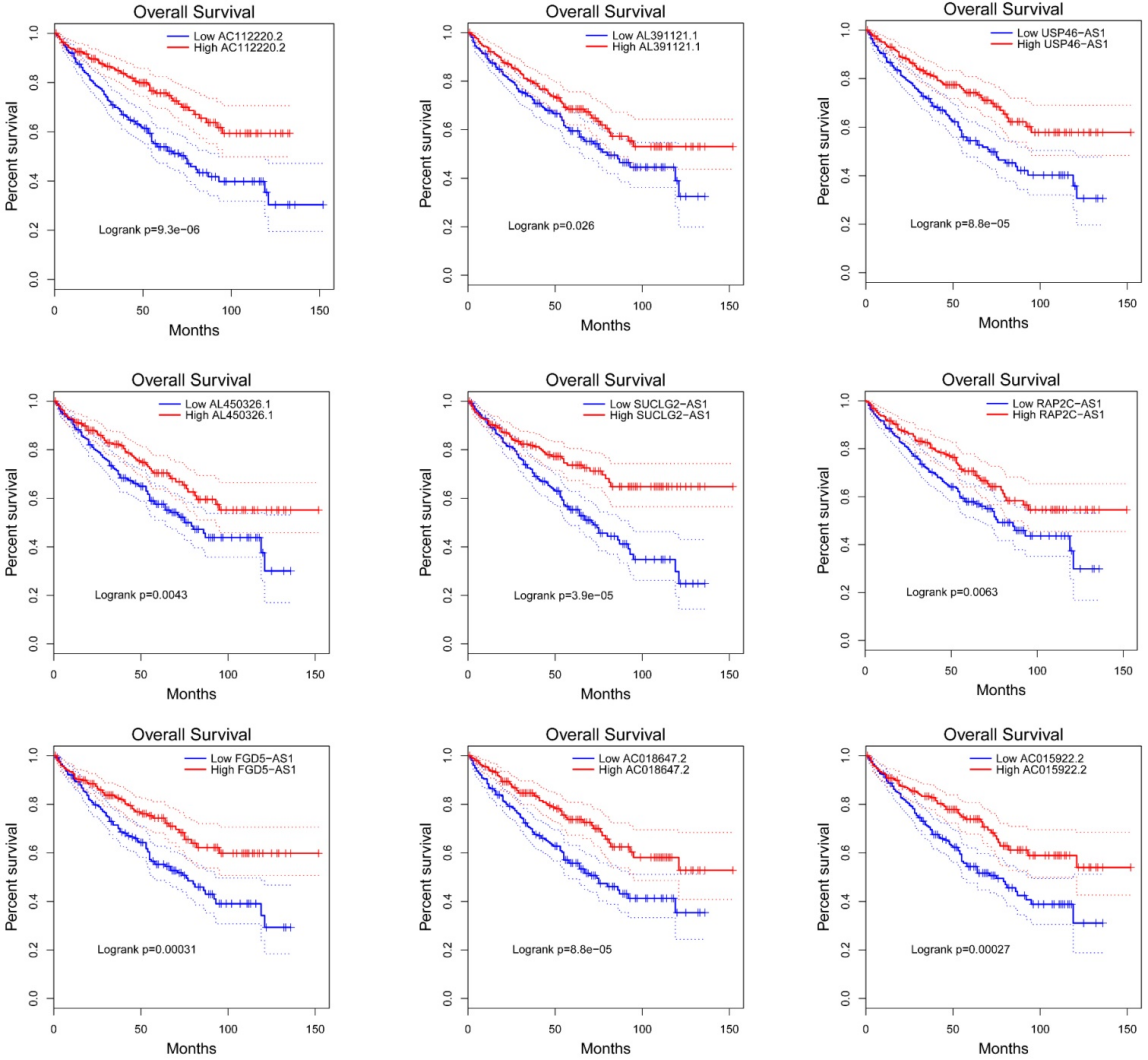
** Kaplan-Meier curves of OS in patients with ccRCC.** Patients were grouped according to the median cutoff of AC112220.2, AL391121.1, USP46-AS1, AL450326.1, SUCLG2-AS1, RAP2C-AS1, FGD5-AS1, AC018647.2 and AC015922.2 expression for OS detection. Patients were separated into two groups (low expression group (n=268) and high expression group (269)) according to the median cutoff of lncRNAs expression.

**Figure 5:**
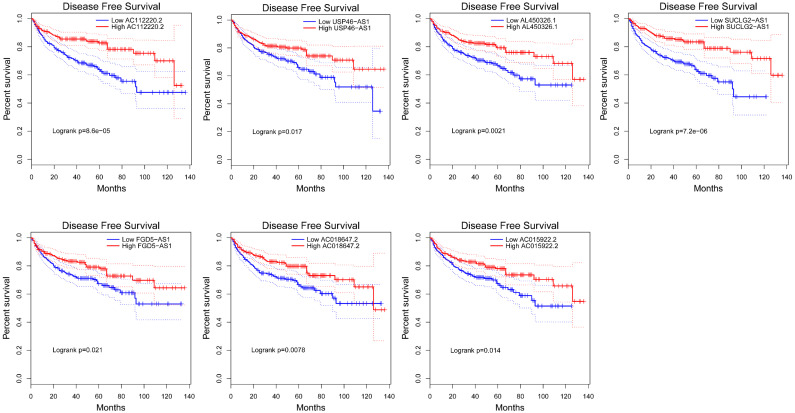
** Kaplan-Meier curves of DFS in patients with ccRCC.** Patients were grouped according to the median cutoff of AC112220.2, USP46-AS1, AL450326.1, SUCLG2-AS1, FGD5-AS1, AC018647.2 and AC015922.2 expression for DFS detection. Patients were separated into two groups (low expression group (n=245) and high expression group (n=246)) according to the median cutoff of lncRNAs expression.

To explore the prognostic characteristics of VHL-related lncRNAs, we analyzed the effects of these lncRNAs on the OS and DFS among ccRCC patients by generating Kaplan-Meier survival curves. Results of the log-rank test showed that the high expression groups of AC112220.2, AL391121.1, USP46-AS1, AL450326.1, SUCLG2-AS1, RAP2C-AS1, FGD5-AS1, AC018647.2 and AC015922.2 had significantly longer OS than their respective low expression groups (Figure 4). Meanwhile, results of the log-rank test also showed that the high AC112220.2, USP46-AS1, AL450326.1, SUCLG2-AS1, FGD5-AS1, AC018647.2 and AC015922.2 expression groups had remarkably longer DFS than their respective low expression groups (Figure 5).

### Association between VHL-related lncRNAs and the clinicopathological characteristics of ccRCC

**Figure 6:**
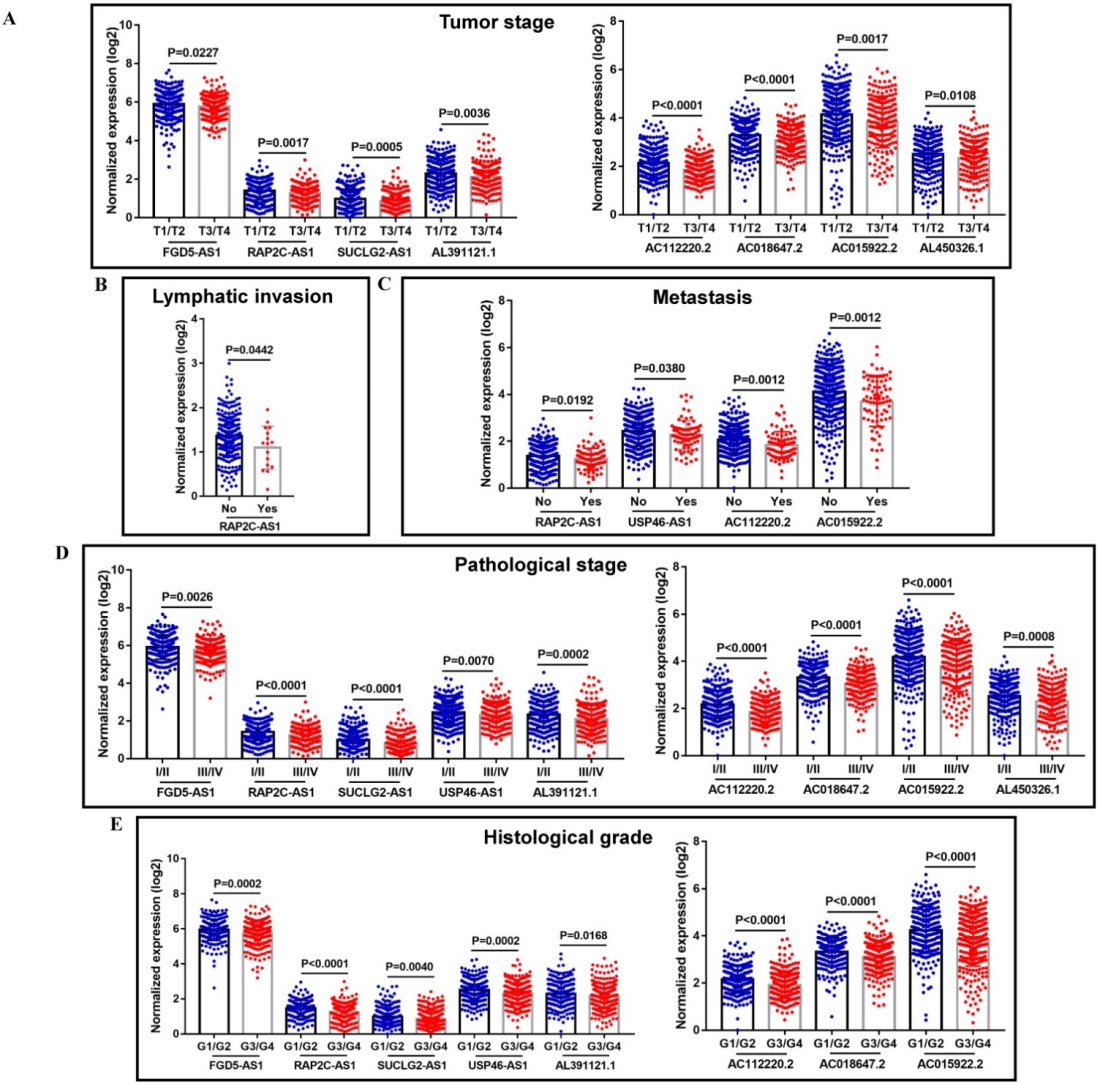
** The correlation between VHL-related lncRNAs expression and the clinicopathological characteristics of ccRCC patients.** The correlation between VHL-related lncRNAs expression and tumor stage (A), lymphatic invasion (B), metastasis (C), pathological stage (D) and histological grade (E).

When concerning the correlation between these VHL-related lncRNAs and the progression of ccRCC, most of them were closely related to some clinical parameters of ccRCC, including tumor stage, lymphatic invasion, metastasis, pathological stage and histological grade. The detailed clinical information of TCGA-KIRC patients was summarized in Supplemental Table 2. Lower expression of FGD5-AS1, RAP2C-AS1, SUCLG2-AS1, AL391121.1, AC112220.2, AC018647.2, AC015922.2 and AL450326.1 were associated with advanced tumor stage (Figure 6A); lower expression of RAP2C-AS1 was related to lymphatic invasion (Figure 6B); lower expression of RAP2C-AS1, USP46-AS1, AC112220.2 and AC015922.2 tended to promote tumor metastasis (Figure 6C); lower FGD5-AS1, RAP2C-AS1, SUCLG2-AS1, USP46-AS1, AL391121.1, AC112220.2, AC018647.2, AC015922.2 and AL450326.1 expression levels were correlated with higher pathological stage (Figure 6D); lower FGD5-AS1, RAP2C-AS1, SUCLG2-AS1, USP46-AS1, AL391121.1, AC112220.2, AC018647.2 and AC015922.2 expression levels were also correlated with higher histological grade (Figure 6E).

### Prognostic and clinicopathological characteristics of VHL-related lncRNAs in ccRCC with VHL mutation

**Figure 7:**
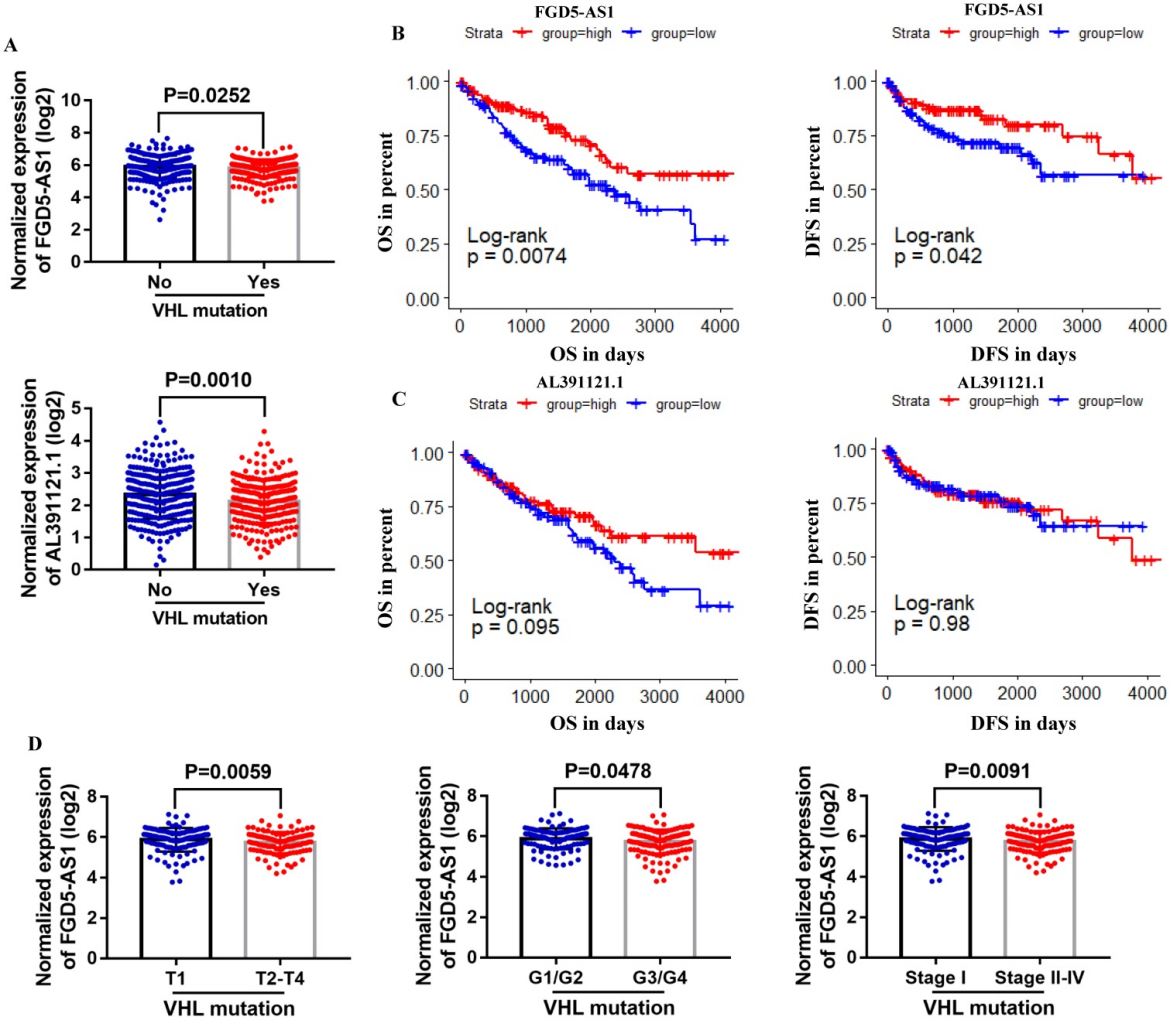
** Comparison of lncRNAs expression in VHL mutant and non-mutant ccRCC tissues.** (A) FGD5-AS1 and AL391121.1 expression in VHL mutant and non-mutant ccRCC tissues. (B and C) Patients were grouped according to the median cutoff of FGD5-AS1 and AL391121.1 expression for OS and DFS detection. (D) Comparison of FGD5-AS1 expression in different tumor stage, historical grade and pathological stage.

To further explore the correlation between these lncRNAs and VHL, we compared their expression in VHL mutant and non-mutant ccRCC tissues. Results suggested that only FGD5-AS1 and AL391121.1 expression were decreased in VHL mutant tissues compared with VHL non-mutant tissues (Figure 7A). Survival analysis results indicated that high expression group of FGD5-AS1 had significantly longer OS and DFS than their respective low expression groups in VHL mutant ccRCC (Figure 7B), while there was no difference in survival time between the high AL391121.1 expression group and its respective low expression group (Figure 7C). Besides, lower FGD5-AS1 expression was related to advanced tumor stage and higher histological grade and pathological stage (Figure 7D).

### FGD5-AS1 expression were negatively correlated with their DNA methylation status in ccRCC

**Figure 8:**
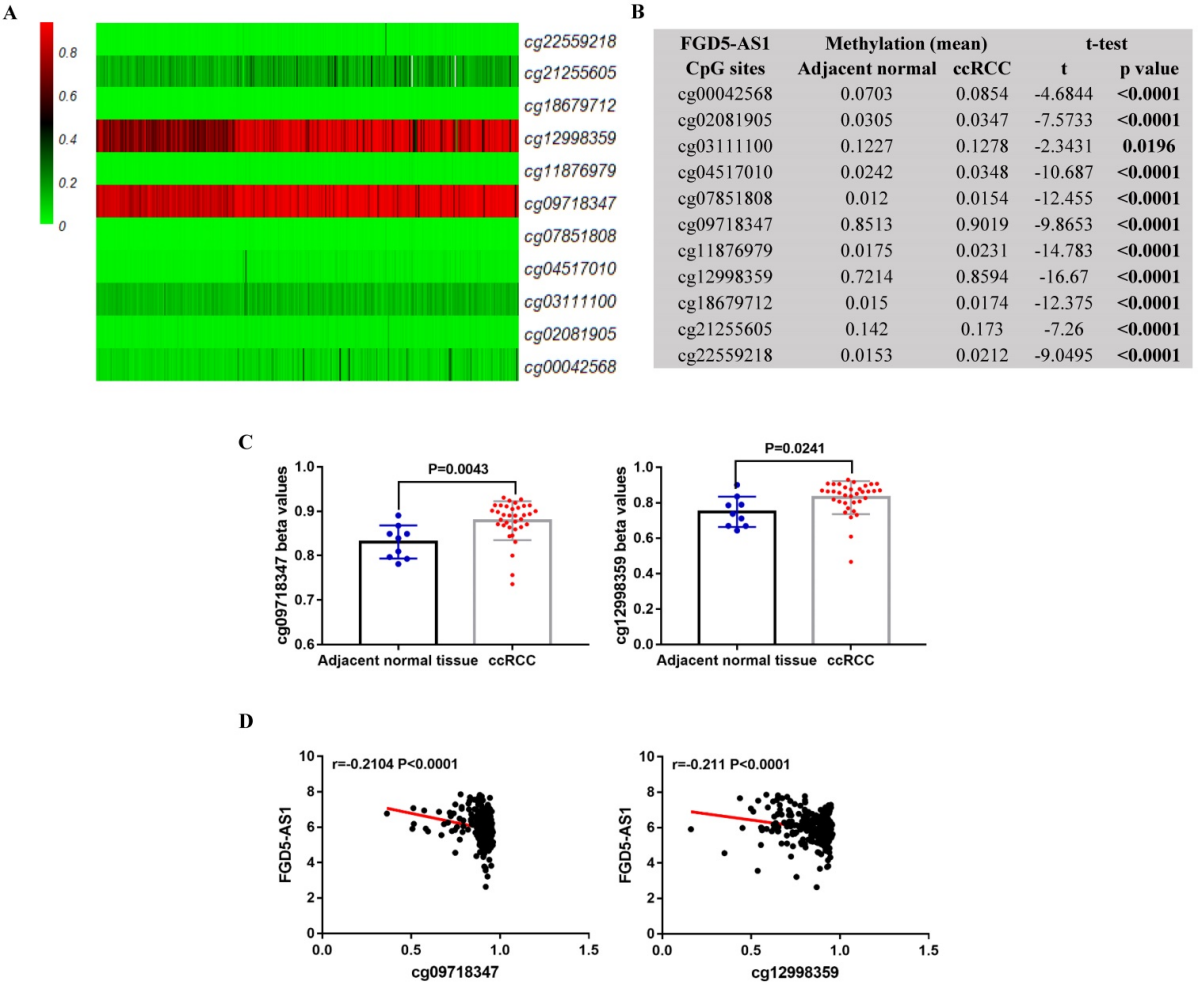
** FGD5-AS1 expression was associated with its DNA methylation status.** Heatmap (A) and statistical comparison (B) of the difference in methylation levels in 11 CpG sites of FGD5-AS1. (C) Validation of the methylation levels of cg09718347 and cg12998359 based on GSE105260 dataset. (D) The correlation between FGD5-AS1 expression and the methylation status of its CpG sites.

In TCGA-KIRC, 325 ccRCC tissues samples and 160 adjacent normal tissues samples were subjected to DNA methylation analysis simultaneously. Using the methylation data, we compared the methylation status of 11 CpG sites (cg00042568, cg02081905, cg03111100, cg04517010, cg07851808, cg09718347, cg11876979, cg12998359, cg18679712, cg21255605 and cg22559218) in FGD5-AS1 DNA, and the detailed information of these CpG sites was provided in Table 2. Results showed that these CpG sites were significantly hypermethylated in the ccRCC tissues compared to adjacent normal tissues (Figure 8A and 8B). Moreover, the data from GEO validated the higher methylation levels of cg09718347 and cg12998359 in the ccRCC tissues compared to adjacent normal tissues (Figure 8C). To further examine the potential regulatory effect of DNA methylation on FGD5-AS1, we analyzed the correlation between FGD5-AS1 expression and the methylation status of their CpG sites. Correlation analysis results showed that the expression of FGD5-AS1 were negatively associated with the methylation levels of cg09718347 and cg12998359 (Figure 8D).

### Validation of the expression of FGD5-AS1 in VHL mutant and non-mutant ccRCC tissues and cell lines

**Figure 9:**
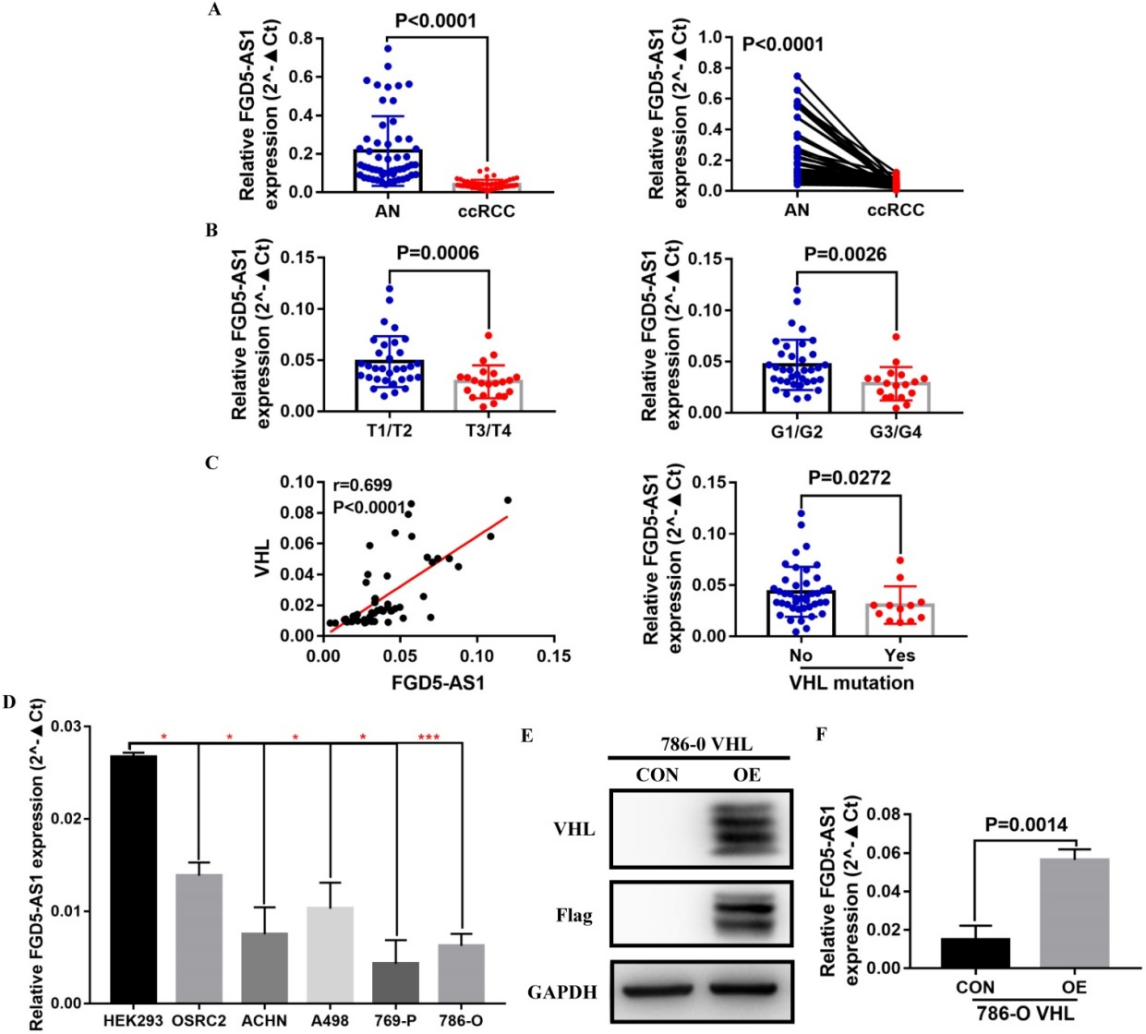
** Validation of the expression of FGD5-AS1 in VHL mutant and non-mutant ccRCC tissues and cell lines.** (A) FGD5-AS1 expression in 54 paired clinical ccRCC tissues. (B) Comparison of FGD5-AS1 expression in different tumor stage and histological grade. (C) Correlation between FGD5-AS1 expression and VHL expression. (D) FGD5-AS1 expression in VHL mutant and non-mutant RCC cell lines, *p<0.05, ***p<0.001 vs. HEK293. (E) VHL protein was successfully overexpressed in 786-O cells. (F) FGD5-AS1 expression was increased in VHL-overexpressed 786-O cells.

To validate the correlation between FGD5-AS1 and VHL expression, we examined the expression of FGD5-AS1 and VHL in 12 VHL mutant and 42 non-mutant ccRCC tissues, and detailed clinical and pathological data of these 54 ccRCC patients were provided in Table 3. Our results confirmed that FGD5-AS1 expression was decreased in these 54 ccRCC tissues compared with their adjacent normal tissues (Figure 9A). Besides, FGD5-AS1 expression was lower in advanced tumor stage and higher histological grade (Figure 9B). Moreover, FGD5-AS1 expression was highly positively correlated with VHL expression, and FGD5-AS1 expression was significantly reduced in VHL mutant ccRCC tissues compared to VHL non-mutant ccRCC tissues (Figure 9C). In addition, we examined the expression of FGD5-AS1 in HEK293 cell line, VHL wild-type RCC cell lines (OSRC2, ACHN and A498) and VHL mutant RCC cell lines (769-P and 786-O). Results indicated that FGD5-AS1 expression was decreased in these RCC cell lines compared with HEK293 cell line, especially in 769-P and 786-O cell lines (Figure 9D). Furthermore, we found that FGD5-AS1 expression was increased in VHL-overexpressed 786-O cells compared with the control 786-O cells (Figure 9E, F).

## Discussion

It is all known that VHL play a critical tumor suppressor role in ccRCC, and VHL gene inactivation is by far the most common carcinogenic driving event in ccRCC. Although lncRNAs play important roles in the development and progression of ccRCC 16, 17, there are currently few literatures on the role of VHL-related lncRNAs in ccRCC.

In this study, we found that several lncRNAs, including FGD5-AS1, RAP2C-AS1, SUCLG2-AS1, USP46-AS1, AL391121.1, ENSG00000271020, AC112220.2, AC018647.2, AC015922.2, MID1IP1-AS1, AL731577.2 and AL450326.1 were co-expressed with VHL, and their expression were positively correlated with VHL expression. Intriguingly, all of them were down-regulated in ccRCC tissues compared to adjacent normal tissues. Besides, the analysis results of catRAPID showed that AC112220.2, AL391121.1, USP46-AS1, AL450326.1, MID1IP1-AS1, SUCLG2-AS1, RAP2C-AS1, FGD5-AS1, AC018647.2 and AC015922.2 have a certain binding potential with VHL protein. Moreover, results of the log-rank test showed that high expression groups of AC112220.2, AL391121.1, USP46-AS1, AL450326.1, SUCLG2-AS1, RAP2C-AS1, FGD5-AS1, AC018647.2 and AC015922.2 had significantly longer OS than their respective low expression groups, and high AC112220.2, USP46-AS1, AL450326.1, SUCLG2-AS1, FGD5-AS1, AC018647.2 and AC015922.2 expression groups had remarkably longer DFS than their respective low expression groups. In addition, they were also closely associated with some clinical parameters of ccRCC, including tumor stage, metastasis, pathological stage and histological grade.

To further explore the correlation between these lncRNAs and VHL, we compared their expression in VHL mutant and non-mutant ccRCC tissues. Results showed that FGD5-AS1 and AL391121.1 expression were remarkably decreased in VHL mutant tissues compared with VHL non-mutant tissues. However, only high expression group of FGD5-AS1 had significantly longer OS and DFS than its respective low expression groups in VHL mutant ccRCC. Besides, we validated the lower expression of FGD5-AS1 in VHL mutant and non-mutant ccRCC tissues and cell lines. Moreover, we found that FGD5-AS1 expression was increased in VHL-overexpressed 786-O cells compared with the control 786-O cells.

Epigenetic alterations have been identified as one of the hallmarks of tumorigenesis 18, 19, and epigenetic regulation is one of the main mechanisms utilized to control lncRNAs expression and tissue specificity 20-22. For instance, survival-related lncRNAs SNHG12 and MINCR were epigenetically activated in multiple cancer types, including breast, bladder, endometrial, colorectal, and lung cancer 23; EPIC1 was epigenetically activated and correlated with poor survival in breast cancer 24; DNA-methylation-mediated activating of lncRNA SNHG12 promoted temozolomide resistance in glioblastoma 25. Moreover, a recent study indicated that SNHG3 and SNHG15 were valuable prognostic markers for ccRCC, and DNA hypomethylation might play an important role in increased SNHG3 and SNHG15 expression in ccRCC 26. In this study, we also examined the correlation between FGD5-AS1 expression and its DNA methylation status. Results showed that the methylation status of cg09718347 and cg12998359 might substantially influence FGD5-AS1 expression in ccRCC.

Previous studies have identified the important roles of FGD5-AS1 in several types of human cancer, including colorectal cancer 27, oral cancer 28, non-small cell lung cancer 29 and esophageal squamous cell carcinoma 30. FGD5-AS1 was aberrantly up-regulated in these tumors compared to adjacent normal tissues. Moreover, increased FGD5-AS1 expression manifested a close association with tumor size, TNM stage, and lymph node metastasis in esophageal squamous cell carcinoma 30; FGD5-AS1 may promote NSCLC cell proliferation 29; FGD5-AS1 knockdown inhibited colorectal cancer cell proliferation, migration, and invasion, and promoted cell apoptosis 27. However, in this study, we found that FGD5-AS1 expression was significantly lower in ccRCC than in adjacent normal tissues, and increased FGD5-AS1 was associated with of longer OS and DFS. Moreover, we found that FGD5-AS1 was significantly associated with VHL, and DNA methylation might play an important role in altered FGD5-AS1 expression.

The loss of pVHL (VHL protein) function affects several cellular processes, of which the activation of HIFs (hypoxia inducible factors) pathway is the best-known function. Constitutive activation of HIFs signaling in turn activates hundreds of genes involved in numerous oncogenic pathways that contribute to the development or progression of ccRCC 17. Previous studies reported that hypoxia/HIF-1α-induced lincRNA-p21 is able to bind HIF-1α and pVHL and thus disrupts the VHL-HIF-1α interaction; SNHG11 binds to the pVHL recognition sites on HIF-1α, thus blocking the interaction of pVHL with HIF-1α and preventing its ubiquitination and degradation, resulting in the increased expression of HIF-1α target genes such as AK4, ENO1, HK2 and Twist1 in colorectal cancer 31. Based on these findings, we can infer that VHL binding lncRNAs may affect the interaction between pVHL and HIFs, thereby affecting HIFs and its target genes expression. Therefore, identification of VHL-related lncRNAs and further exploration of their regulatory mechanisms can provide new theoretical basis for tumor therapy.

In summary, we for the first time identified VHL-related lncRNAs through WGCNA, correlation analysis and catRAPID algorithm, and explored their prognostic and clinicopathological characteristics in ccRCC. As a result, we found that FGD5-AS1 was significantly associated with VHL expression and DNA hypermethylation might play an important role in decreased FGD5-AS1 expression. Moreover, FGD5-AS1 can serve as a valuable diagnostic and prognostic markers in ccRCC. To further confirm our findings, more studies are needed to explore the specific relationship between FGD5-AS1 and VHL, and to verify its prognostic value.

## Supplementary Material

Supplementary tables.

## Figures and Tables

**Table 1:** The co-expressed lncRNAs of VHL identified by WGCNA

Gene 1	Gene 2		Weight
	ensembl	gene symbol	
VHL	ENSG00000225733	FGD5-AS1	0.051624798
VHL	ENSG00000232160	RAP2C-AS1	0.043959581
VHL	ENSG00000241316	SUCLG2-AS1	0.032784519
VHL	ENSG00000248866	USP46-AS1	0.027462711
VHL	ENSG00000272933	AL391121.1	0.026646185
VHL	ENSG00000271020	RP11-10C24.1	0.025374632
VHL	ENSG00000271643	AC112220.2	0.024199759
VHL	ENSG00000271122	AC018647.2	0.023547268
VHL	ENSG00000265519	AC015922.2	0.023450544
VHL	ENSG00000238123	MID1IP1-AS1	0.021209168
VHL	ENSG00000249456	AL731577.2	0.020339211
VHL	ENSG00000230555	AL450326.1	0.020245942

**Table 2:** The detailed information of CpG sites of FGD5-AS1 DNA

Composite Element REF	Chromosome	Start	End	CGI_Coordinate	Feature_Type
cg00042568	chr3	14946432	14946433	CGI:chr3:14947105-14948477	N_Shore
cg02081905	chr3	14947162	14947163	CGI:chr3:14947105-14948477	Island
cg03111100	chr3	14948476	14948477	CGI:chr3:14947105-14948477	Island
cg04517010	chr3	14948461	14948462	CGI:chr3:14947105-14948477	Island
cg07851808	chr3	14947328	14947329	CGI:chr3:14947105-14948477	Island
cg09718347	chr3	14933436	14933437	CGI:chr3:14947105-14948477	.
cg11876979	chr3	14947322	14947323	CGI:chr3:14947105-14948477	Island
cg12998359	chr3	14944016	14944017	CGI:chr3:14947105-14948477	N_Shelf
cg18679712	chr3	14948165	14948166	CGI:chr3:14947105-14948477	Island
cg21255605	chr3	14947624	14947625	CGI:chr3:14947105-14948477	Island
cg22559218	chr3	14947874	14947875	CGI:chr3:14947105-14948477	Island

**Table 3:** The clinical and pathological characteristics of the 54 ccRCC patients that used for validation

Clinicopathologic characteristics	N (%)
**Age**	
<60	31 (57.4)
≥60	23 (42.6)
**Gender**	
Male	32 (59.3)
Female	22 (40.7)
**Tumor stage**	
T1	20 (37.0)
T2	12 (22.2)
T3	21 (38.9)
T4	1 (1.9)
**Lymph node stage**	
N0	52 (96.3)
N1	2 (3.7)
**Pathological stage**	
Stage I	22 (40.7)
Stage II	17 (31.5)
Stage III	12 (22.2)
Stage IV	3 (5.6)
**Histological grade**	
G1	18 (33.3)
G2	18 (33.3)
G3	17 (31.5)
G4	1 (1.9)
**VHL mutation**	
No	42 (77.8)
Yes	12 (22.2)

## Abbreviations

ccRCC: clear cell renal cell carcinoma
lncRNAs: long non-coding RNAs
TCGA-KIRC: the Cancer Genome Atlas-Kidney Renal Clear Cell Carcinoma
WGCNA: Weighted Gene Co-expression Network Analysis
OS: Overall Survival
DFS: Disease Free Survival
VHL: Von Hippel-Lindau
pVHL: VHL protein
HIFs: hypoxia inducible factors
