# Supplementary material for: Identification and validation of the clinical roles of the VHL-related LncRNAs in clear cell renal cell carcinoma
Source: J Cancer. 2021 Mar 5;12(9):2702–14. doi: 10.7150/jca.55113 (PMC8040721; doi:10.7150/jca.55113)
Supplement: Supplementary file 1 — Supplementary tables. [file jcav12p2702s1.pdf]

Supplemental Table 1: The detailed primer sequences for q-RT-PCR

q-RT-PCR

Gene	Primer	Sequence (5’-3’)	Nt	Tm	Length of amplicon	GC%
FGD5-AS1	Forward	ACTAGAAGCGGAGGGGTGAA	20	60.25	126	55
	Reverse	CGCCATTTTGTTCTCGACGG	20	60.18		55
GAPDH	Forward	GAAAGCCTGCCGGTGACTAA	20	60.32	113	55
	Reverse	GCATCACCCGGAGGAGAAAT	20	59.82		55

Supplemental Table 2: The detailed clinical information of TCGA-KIRC patients

Clinicopathologic characteristics		N(%)
Age		
	<60	247 (46.0)
	>=60	290 (54.0)
Gender		
	Male	346 (64.4)
	Female	191 (35.6)
Tumor stage		
	T1	275 (51.2)
	T2	69 (12.8)
	T3	182 (33.9)
	T4	11 (2.1)
Metastasis		
	No	446 (84.6)
	Yes	81 (15.4)
Lymph node stage		
	N0	240 (44.7)
	N1	17 (3.2)
	NX	280 (52.1)
Pathological stage		
	Stage I	269 (50.3)
	Stage II	57 (10.6)
	Stage III	125 (23.4)
	Stage IV	84 (15.7)
Histological grade		
	G1	14 (2.7)
	G2	230 (43.5)
	G3	207 (39.1)
	G4	78 (14.7)
VHL mutation		
	No	284 (53.7)
	Yes	245 (46.3)
